# Deadly acute Decompression Sickness in Risso’s dolphins

**DOI:** 10.1038/s41598-017-14038-z

**Published:** 2017-10-19

**Authors:** A. Fernández, E. Sierra, J. Díaz-Delgado, S. Sacchini, Y. Sánchez-Paz, C. Suárez-Santana, M. Arregui, M. Arbelo, Y. Bernaldo de Quirós

**Affiliations:** Veterinary Histology and Pathology, Institute of Animal Health, University of Las Palmas de Gran Canaria, Veterinary School, C/Transmontaña s/n, 35416 Arucas, Las Palmas Spain

## Abstract

Diving air-breathing vertebrates have long been considered protected against decompression sickness (DCS) through anatomical, physiological, and behavioural adaptations. However, an acute systemic gas and fat embolic syndrome similar to DCS in human divers was described in beaked whales that stranded in temporal and spatial association with military exercises involving high-powered sonar. More recently, DCS has been diagnosed in bycaught sea turtles. Both cases were linked to human activities. Two Risso’s dolphin (*Grampus griseus*) out of 493 necropsied cetaceans stranded in the Canary Islands in a 16-year period (2000–2015), had a severe acute decompression sickness supported by pathological findings and gas analysis. Deadly systemic, inflammatory, infectious, or neoplastic diseases, ship collision, military sonar, fisheries interaction or other type of lethal inducing associated trauma were ruled out. Struggling with a squid during hunting is discussed as the most likely cause of DCS.

## Introduction

Pathologies related to effects of changes in pressure are well known among human divers. Decompression sickness (DCS) is a syndrome related to the formation of gas bubbles in blood and/or tissues when the sum of the dissolved gas tensions exceeds the local absolute pressure. Gas bubbles may have biochemical effects and disrupt the tissues or occlude the vessels with clinical and pathological signs and, in certain cases, death^1^.

Marine mammals have long been considered protected against DCS through anatomical, physiological, and behavioural adaptations^2^. However, an acute systemic gas and fat embolic syndrome similar to DCS in human divers was described in beaked whales that stranded in temporal and spatial association with military exercises involving high-powered sonar^3,4^.

Several hypotheses have been proposed as a cause–effect relationship between mid-frequency active (MFA) sonar use and these stranding events^5^. One of them is the alteration of beaked whales’ diving behaviour in response to MFA sonar exposure in such a manner that behavioural or physiological mechanisms employed for protecting against the formation of N_2_ gas bubbles were overridden^4,5^. According to this proposal, bubble evolution might occur as a result of severe alterations in dive behaviour (e.g., extremely rapid surfacing or remaining at the surface and possibly vigorously swimming)^5^.

During the last decade, there has been accumulating evidence demonstrating the presence of gas bubbles in diving marine mammals^6–11^. Theoretical models have predicted end dive N_2_ tensions sufficient to cause supersaturation of the tissues in marine mammals even under normal diving conditions despite their access to an extensive repertoire of adaptations to mitigate gas loading^12–14^. The composition of gas bubbles in bycaught marine mammals proved to come from off gassing N_2_ saturated tissues^11^. In addition, DCS has been diagnosed clinically and pathologically in bycaught sea turtles^15^, linked also to human activities.

From 2000 to 2015, 506 cetaceans stranded and died or died and stranded in the Canary Islands (Spain). Systematic pathological studies were performed on the carcasses to find out the cause of death and/or stranding. Of those, 13 were beaked whales stranded in temporal and spatial association with military exercises^4,16^. Within the remaining 493 cetaceans, we found two more cases with similar lesions as those described previously in beaked whales^3,4^. Unlike in beaked whales stranded in temporal and spatial association with military exercises, the evidence in these two cases supported a natural cause as the disturbance triggering fatal systemic gas embolism.

In this manuscript we describe pathological findings and gas analysis consistent with acute DCS in two stranded Risso´s dolphins (*Grampus griseus*), together with evidence of stressful and lethal predatory interactions with a squid.

## Results

During the study period (2000–2015), two Risso’s dolphins presented pathological findings and gas analysis consistent with DCS. Pathological studies were performed in ten more Risso’s dolphins within the study period. A summary of the biological and pathological data of the twelve dolphins is shown in Table 1 for comparison purposes. Below we describe in detail the necropsy, histological and ancillary laboratory results for the two cases with DCS together with evidence of lethal predatory interactions with squid.

**Table 1:** List of Risso’s dolphins with complete pathological studies within the study period (2000–2015).

Decomposition code	Case No	Stranding Date	Age	Sex	Found	Body condition	Gas Score	Esophagus content	Stomach content	Pathology
1	CET 431	21/04/08	Juvenile	M	A	Poor	1	Empty	Some squid beaks and otolites, many anisakis and FB	Encephalitis
2	CET 456	17/06/08	Adult	F	A	Moderate	2	Empty	Many squid beaks.	Meningoencephaltis
	CET 472	7/11/08	Calf	F	D	Very poor	1	Empty	Many squid beaks. Few FB.	Fishing interaction/Trauma
	*CET* 483 *case* 1	*6/3/09*	*Subadult*	*M*	*D*	*Good*	7*	Squid tentacles from mouth to stomach. Hemorragic lesions.	*Body of one large intact squid*, *and body parts of 4 small squids*	*Gas embolic pathology*
	CET 534	22/04/10	Subadult	M	D	Poor	3	Sand	Sand. 3 squid beaks. FB.	Chronic multiorganic inflamatory pathologies
	*CET* 549 *case* 2	*14/09/*10	*Adult*	*F*	*D*	*Moderate*	*10*	*Empty Ulcers*	*One very large intact squid distending the stomach*	*Gas embolic pathology*
	CET 578	29/05/11	Adult	F	A	Poor	2	Empty	Many squid beaks. Some anisakis.	Chronic multiorganic immflamatory pathologies and non supurative meningoencephalitis
3	CET 199	19/01/03	Adult	M	D	Good	—	Empty	Few squid beaks and otolites. Nematodes.	Severe Pneumonia
4	CET 533	20/04/10	Adult	M	D	Poor	7	Anisakis	One large intact squid. Food remains. Many squid beaks. Many anisakis.	Not determined.
	CET 565	3/22/11	Subadult	F	D	Poor	4*	Few shellfish	Many squid beaks. Few nematodes	Chronic multiorganic immflamatory pathologies
	CET 634	11/3/12	Adult	M	D	Poor	—	Sand and algae	FB	Severe parasitic sinusitis and stomach foreign body ingestion
	CET 751	16/03/15	Adult	F	D	Good	8*	Brown mucous substance	Empty	Parasitic chronic sinusitis

Asterisks indicate dolphins on which one localization was not adequately observed for bubbles, thus the gas score could potentially be up to 2 points higher in these animals if bubbles were present on that location.

### Necropsy and histological findings

#### Case 1 (CET 483)

**Figure 1:**
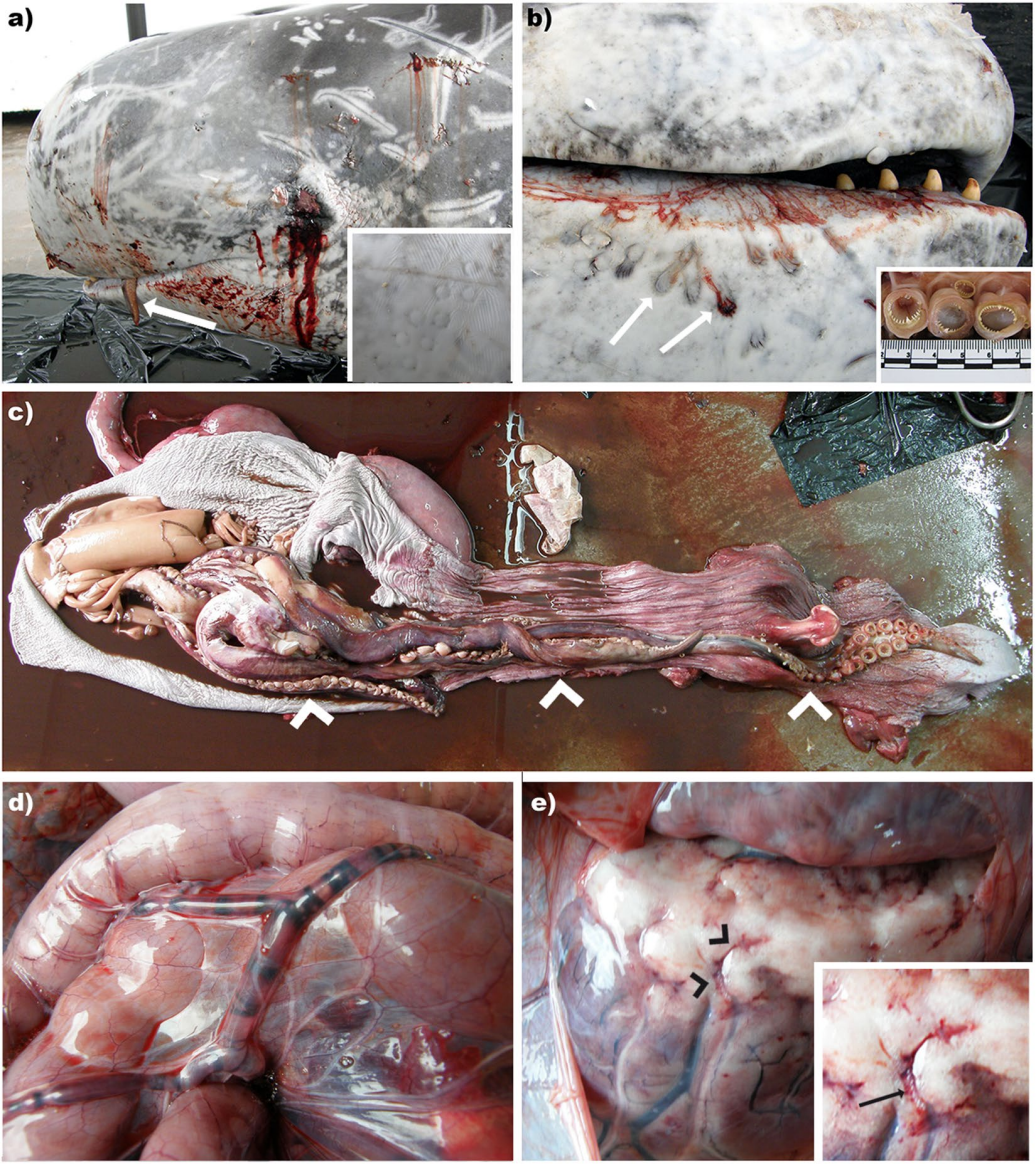
Gross findings of Case 1. (**a**) Squid tentacle partially fixed to the mandible (arrow). Inset: Squid tentacle suckers’ impressions on the cervical skin. (**b**) Mild multifocal and superficial ring-shape cutaneous lacerations caused by squid’s tentacle suckers and hooks (arrows). Inset: gross detail of squid’s tentacle suckers and hooks. (**c**) Upper digestive tract content of case 1, consisting in an entire squid along the oropharynx, oesophagus, and stomach (arrowheads). (**d**) GFVD in the mesenteric veins. (**e**) Mild multifocal haemorrhages following the vascular tracts (arrowheads). Inset: GFVD in the coronary veins associated to petechial haemorrhages (arrow).

A 295 cm-long, male, Risso’s dolphin was observed swimming randomly near the coast of Fuerteventura on March 6, 2009. The animal was found later on the same day stranded dead very fresh (code 1) and in good body condition. Necropsy was performed within 24 hours post-mortem. Externally, signs consistent with a live stranding as well as intra- and interspecific interaction marks were observed. The distal segment of a squid tentacle endowed with suckers and rings with hooks was observed partially fixed to the mandibular skin (Fig. 1a,b,c). Additional superficial squid sucker and ring-associated marks of varying depth were observed throughout the cervical skin (Fig. 1a). On necropsy examination, the main finding was abundant systemic gas-filled vascular dilations (gas bubbles; GFVD) in the mesenteric (Fig. 1d), epigastric, splenic, diaphragmatic, inter-renicular veins as well as in the lumbo-caudal plexus. In the mesenteric veins, gas bubbles coalesced and obliterated large vessel segments. Extravascular gas bubbles were also grossly detected beneath the renal capsule and in the heart coronary fat depots, typically associated with petechial haemorrhages (Fig. 1e).

In the digestive tract, two large squid tentacles (110 cm-long) ran through the oesophagus (Fig. 1c). The cranial part of the oesophagus had focal acute haemorrhages associated with tentacle’s suckers and hooks. An intact large undigested squid and additional partially digested squid arms, head and mantels were present in the lumen of the keratinized gastric compartment mixed with abundant dark liquid (Fig. 1c). The squids were identified as *Ommastrephes bartramii* (LeSueur, 1821). Other gross findings were non-collapsed lungs with rib marks, marked pleural lymphangiectasia, a 4 cm focal emphysematous bulla laterally on the cranial right lung, marked pulmonary oedema filling the trachea, and mediastinal lymphadenomegaly and oedema. Examination of the central nervous system (CNS) revealed subjective softness of the cerebrum and cerebellum on palpation.

**Figure 2:**
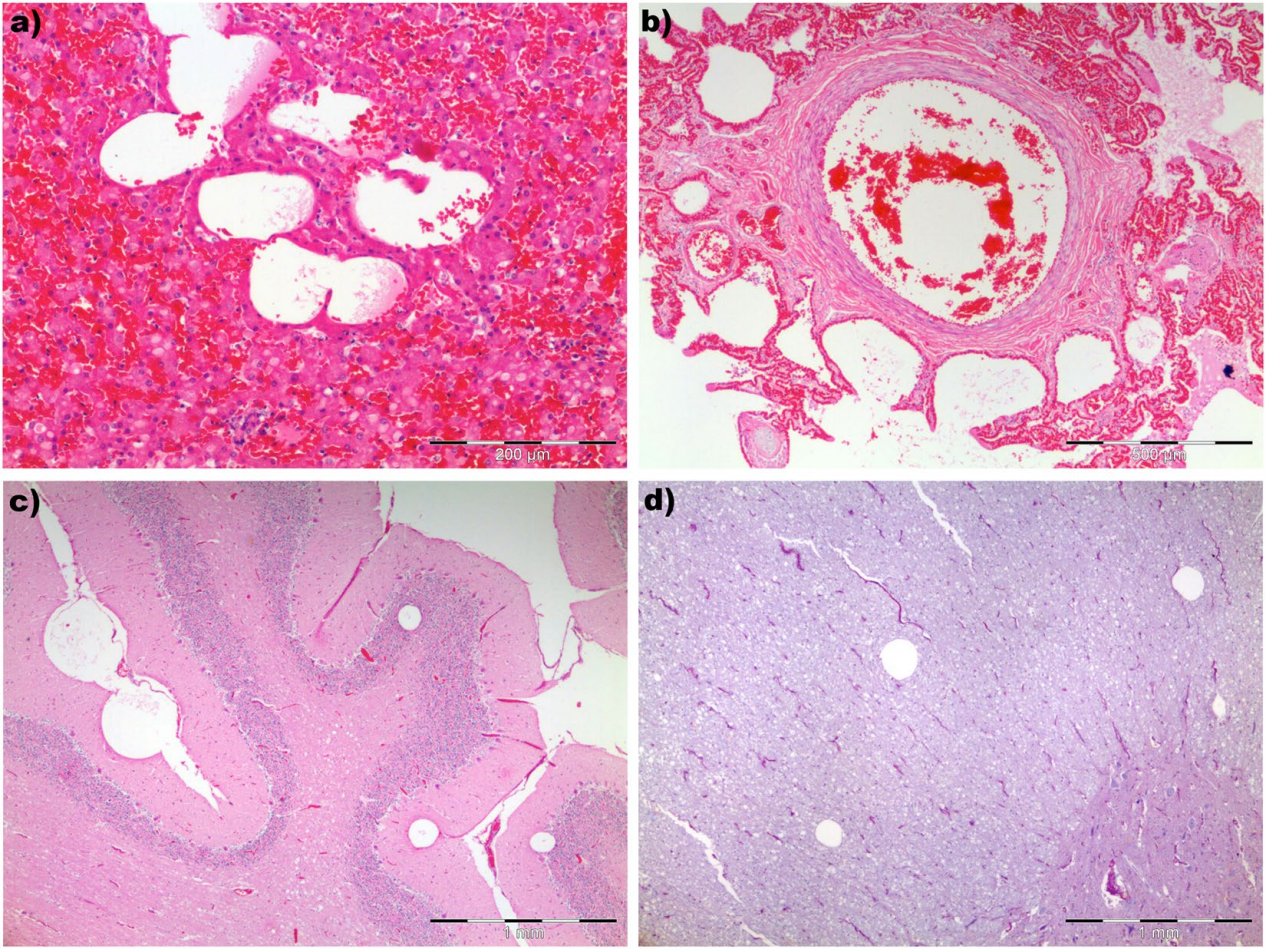
Histopathological findings in two Risso’s dolphins with acute decompressive pathology. Round non-staining spaces among groups of red cells consistent with gas bubbles in liver (**a**), lung (**b**) and cerebellum (**c**). Scattered gas bubbles in the white matter neuroparenchyma of the spinal cord (**d**).

The main histological finding was the presence of multiple small round to oval non-staining spaces (OsO_4_ negative) often displacing groups of erythrocytes consistent with gas bubbles in all tissues examined, but more prominently in renal capillaries and subcapsular veins, hepatic sinusoids (Fig. 2a), pulmonary vessels (Fig. 2b), intestinal submucosal veins and meningeal veins (Fig. 2c). Additional vascular rhexis associated with large, focally expansive intraparenchymal gas bubbles were observed in the white matter of different brain areas as well as in the spinal cord (Fig. 2d). Focal oesophageal haemorrhage and acute myodegeneration and necrosis of the longitudinal and circumferential muscle layers were seen in the injured oesophagus. Additional histological findings included systemic venous congestion, multifocal pulmonary emphysema and diffuse oedema with alveolar histiocytosis, low-grade pulmonary fat embolism^17^, intracytoplasmic eosinophilic globules in hepatocytes, multifocal white matter spongiosus, especially marked in the brainstem, accompanied by proteinaceous perivascular oedema and swollen glial cells. The animal was sexually immature attending to histological features of the testes.

#### Case 2 (CET 549)

**Figure 3:**
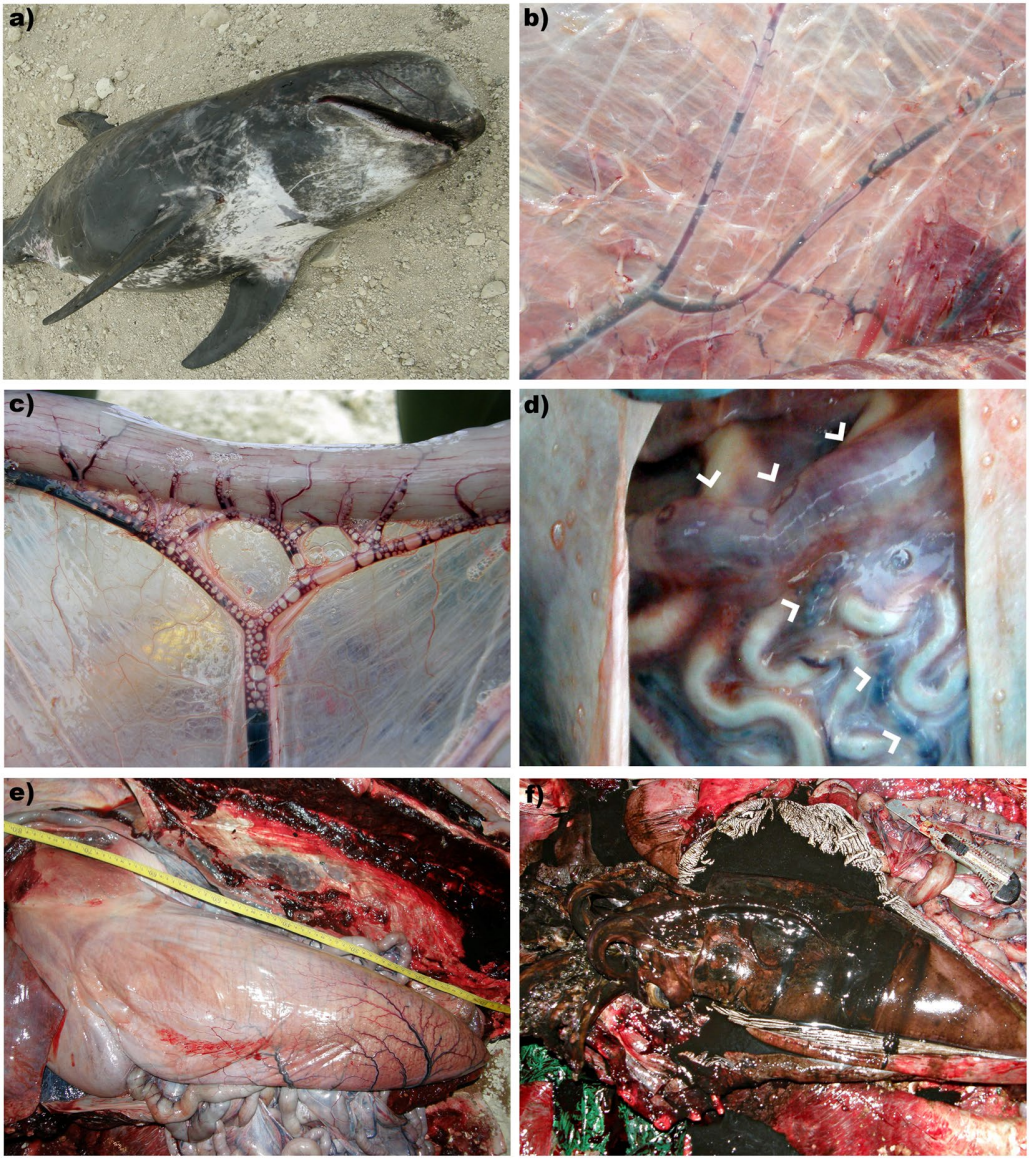
Gross findings of Case 2. (**a**) External view of the animal. (**b**) GFVD in the subcutaneous veins. (**c**) Distended stomach (**d**) opened stomach showing an entire intact squid. (**e**) GFVD in the mesenteric veins. (**f**) GFVD in the coronary veins (arrow heads).

A 240 kg, adult (280 cm-long), female Risso’s dolphin was found stranded dead in Güimar, Tenerife (Spain) on the morning of 14/09/2010^16^. The animal was fresh (code 2) and in poor body condition at necropsy (Fig. 3a). Externally, cutaneous lacerations and linear incisions in the skin as well as scapulo-humeral hemarthros indicated live stranding.

On necropsy examination, main gross finding was abundant systemic GFVD in subcutaneous veins (Fig. 3b), in the venous system in the subcutis and interfascicular fascial planes, subscapularis vein and its tributaries veins, peritoneal, mesenteric (Fig. 3c), diaphragmatic, coronary (Fig. 3d), interventricular, pleural, cerebral, and cervical epidural vasculature. These gas-filled vascular dilations were associated with haemorrhages involving grossly visible small-calibre vessels. The regional lymphatics, and to a lesser extent the arterial system, also presented GFVD^18^. The left lung and aorta were partially collapsed, indicating a possible pneumothorax. In the digestive tract, focal erosions were detected on the tongue and the oesophagus. The fore stomach was distended (Fig. 3e). A large, intact, undigested squid was found in the keratinized gastric compartment (Fig. 3f). Other gross findings included a 5 cm diameter area of lung rupture on the dorsal aspect of the left lobe with marginal fibrosis, mild haemorrhage and extruding gas bubbles, mesenteric and thoracic lymphadenomegaly, mild hydropericardium, and mild systemic parasitism^18^.

The main histological finding was the presence of multiple small round non-staining spaces among groups of red cells consistent with gas bubbles in all organs examined, but more prominently in the mediastinal, pulmonary, mesenteric and periaortic lymph nodes, spleen, trachea, pleura, rete mirabile (thoracic and epidural), pituitary gland, meninges and cerebral and cerebellar neuroparenchyma, occasionally associated with congestion, perivascular oedema and haemorrhages (Fig. 2)^18^.

Other histological findings included the presence of *Sarcocystis* sp., in myofibers of dorso-epaxial muscles, tongue, oesophagus, myocardium and diaphragm; multifocal subendocardial fibrosis; and chronic parasitic enteritis, pericolangitis, hepatitis and pancreatitis. In the lung, atelectatic areas close to emphysematous subpleural foci were observed. The lung-associated pleural injury was microscopically built up of healing processes with neovascularization surrounding empty round spaces (bullous-like). The lungs were negative for fat emboli staining.

### Microbiological and virological results

All samples from Cases 1 and 2 tested negative for Cetacean Morbillivirus (CeMV) and herpesvirus. Bacteriological analyses performed on those same tissues were negative for possible pathogenic recognized bacteria.

### Gas score

Gas score^8^ was calculated for 10 out of 12 Risso’s dolphins. Case 2 (CET 549) presented the highest score reaching the maximum possible scale of the method used^8^. This indicates that all screened veins presented large quantities of gas with some sections completely filled with gas. Case 1 (CET 483) presented as well a very high gas score; although one location could not be evaluated. Both animals presented clearly higher gas score compared to other Risso’s dolphins within their same decomposition code (Table 1).

### Gas analysis

Case 1 (CET 483) gas embolism was composed of 42–52% N_2_ and 39–51% CO_2_ while Case 2 (CET 549) gas embolism was composed of 61–87% N_2_, 8–31% of CO_2_ and 0–14% of O_2_. Gas analysis from the pleural cavity of case 2 confirmed the presence of pneumothorax (84% N_2_, 16% CO_2_).

## Discussion

Of 493 necropsies systematically performed on stranded cetaceans in the Canary Islands, including 26 species of whales and dolphins, only two fresh Risso’s dolphins with different body condition (good and poor, respectively), showed pathological findings and gas analyses consistent with decompression sickness (DCS) as it has been described in naturally occurring cases involving human divers^19,20^ experimental settings in laboratory animals^21,22^, stranded beaked whales linked to navy military sonar^3,4^, and by-caught turtles^15^. To the author’s knowledge, this is the first report of decompression pathology in marine mammals not related to anthropogenic activities and with predation postulated as the most likely disturbance triggering DCS.

The gross (necropsy), histopathology and ancillary laboratory analyses reported in this study indicated that the dolphins had no deadly systemic, inflammatory, infectious, or neoplastic diseases, without external and internal lesions of ship collision or other type of lethal inducing associated trauma (e.g.: fisheries, inter- or intraspecific interactions with other marine animals, excepting squids).

Both dolphins showed large amounts of intra- and extravascular gas bubbles widely distributed, interpreted as an acute systemic gas embolism similar to human divers and experimental animals with acute DCS. The amount of gas observed grossly was higher in these two dolphins than any other Risso’s dolphin within the same decomposition code (Table 1). Case 1, presented the highest gas score (as an index to represent gas abundance and distribution)^23^ in a sub-study of 88 cetaceans belonging to 18 different species stranded in the Canary Islands between 2006 and 2010^8^.

Hydrogen, a putrefaction marker^21,24,25^, was only found in the mesenteric veins of case 1, but absent in the left and right heart ventricles. Hydrogen was absent in all samples from case 2. Thus, gas composition analyses ruled out putrefaction gases as the origin of the gas embolism. In contrast, gas composition was consistent with gases produced by decompression in both dolphins^21^; N_2_ was the main constituent of the gas bubbles. This finding strongly supports the hypothesis that both animals developed a high amount of N_2_ gas bubbles systemically distributed within and outside of the venous system as it is described in DCS^1,19,26^.

The elevated levels of CO_2_ found in case 1 have also been reported experimentally in rabbits that died after a hyperbaric treatment^21^ and in humans with high-magnitude vital gas embolism^20^. These high levels of CO_2_ might indicate an overlapped asphyxiation process during the predator – prey interaction as part of the hypoxic pathogenic mechanisms leading to death.

Acute gas embolism has been described before in beaked whales stranded in temporal and spatial association with military exercises where high intensity mid-frequency sonar was used^3,4^. In the cases presented in this manuscript, military sonar was ruled out since there is a sonar ban established in the Canary waters before these strandings occurred^27^.

In addition to the systemic gas embolism and extravascular gas bubbles similar to human divers or experimental animals with acute DCS, the two dolphins shared one more finding: the presence of large undigested squids in the digestive tract with *intra vitae* associated lesions in the oesophagus. Moreover, in one of the dolphins fight marks could be clearly observed externally.

Risso’s dolphins inhabit deep oceanic and use mostly the upper continental slope waters, generally 400–1,000 m deep^28^, where they are thought to feed on cephalopods^28,29^. The neon flying squid *Ommastrephes bartramii* (LeSueur, 1821) is one of its prey. It is distributed worldwide in subtropical (including Canary Islands) and temperate oceanic waters from the surface until approximately 1500 m depth^30^. The neon flying squid migrates vertically: in subtropical waters they spend the night at shallow depths while they descend to approximately 700 m depth during the day^31^.

In the Canary Islands, there are many species of cetaceans that prey on squids. Remains of squids (mainly beaks) are often found in the stomach of stranded whales and dolphins died due to different causes (Table 1)^32^. Cockcroft *et al*. (1993) found 33 empty stomachs out of 65 stranded Risso’s dolphins. The remaining 32 dolphins presented with cephalopod’s remains, mainly beaks^29^. Blanco *et al*.^29^ found also mainly cephalopod’s beaks in 15 stranded Risso’s dolphins^29^. Bloch *et al*.^33^ found cephalopod’s beaks, and buccal and squid bodies partially digested in 24 Risso’s dolphins that were driven to strand in the Faroe Islands by fishermen. Bloch *et al*.^33^ interpreted these content as an indication that the dolphins had been eating shortly before the drive^33^. In none of the previous cases (0 out of 81) did they report entire indigested squids, or arms running along the oesophagus.

Fresh undigested or partially digested food is a rare finding in stranded cetaceans^29,32^. This finding has been reported associated with fisheries interaction, ship collisions, and military sonar^4,16,32^. In all these cases the death of the animal was acute, preventing the digestion of material.

Ship collision was ruled out as no sign of sharp or blunt trauma was found. Military sonar was also ruled out based on the ban and the epidemiological data as previously discussed. Regarding fisheries interaction, these animals were very fresh and there was an absence of net marks. Additionally, both animals presented signs of active stranding (arriving alive to the shore). Moreover, one of the animals was observed swimming randomly near to shore. It was considered unlikely that these dolphins were beach cast bycaught animals.

The presence of a fresh, entire, and undigested squid filling and protruding from the oral cavity and extending throughout the pharynx, oesophagus and the keratinized gastric compartment was associated with peribuccal and upper digestive tract lesions due to squid suckers in one of the dolphins. This association clearly indicates a fight between the Risso’s dolphin (predator) and the prey (squid) while the dolphin was trying to feed on the squid.

In conclusion, considering the systemic gas embolism, the high N_2_ content of the gas bubbles, and the presence of entire undigested squid in the upper digestive tract and the related lesions, the most plausible explanation is a acute decompression pathology that could have occurred as a result of severe alterations in the diving behaviour and physiology while struggling with the prey during hunting.

Based on the stomach content of the present cases, it is reasonable to consider that during the interaction, the dolphins would enter in a situation characterized by severe stress, abnormal dive behaviour, vigorously swimming, rapid ascending, and struggling at depth and surface. These progressive and accumulating homeostatic disturbances would have overwhelmed anatomical and physiological mechanisms for gas homeostasis^7^ resulting in eventual lethal gas embolism, characterized by widespread sudden appearance of large amounts of intravascular and tissue gas bubbles^1,19^.

Occurrence of systemic gas embolism and lethal prey interaction appears to be very low (2 out of 493 studied cetaceans). Individual risk factors may have influenced the occurrence of systemic gas embolism in these two dolphins. These DCS individual risk factors^34^ may include: (a) level of N_2_ in tissues; (b) hyperlipidaemia (postprandial status); (c) predation experience; (d) pre-existing disease; and (e) stress response. These factors are discussed below.

The number of digested preys found in the keratinized gastric compartment of these animals suggests that they caught their last prey (undigested entire squid), after either a long hunting dive, or after a series of hunting dives. Thus, the animals might have had a high N_2_ tension in the tissues; a key condition for DCS^26^ at the moment of the last prey interaction. A combination of a stressful situation with an altered dive profile and dive response could very likely result in a progressive acute systemic gas embolism (main pathological finding of DCS).

The high concentration of N_2_ could have been aggravated by another decompression risk factor described as “postprandial status” (associated with hyperlipidaemia). Both animals had variably digested food in their stomachs. It is well known that lipids fix five folds more N_2_ than any other tissue^35^. A greater degree of N_2_ supersaturation might decrease the threshold for these putative processes. Fernandez *et al*. (2005) suggested that the postprandial and presumably hyperlipidaemic state of cetaceans in good body condition may favour lipid aggregation in blood following one of the mechanisms described in the fat embolism pathogenesis^36^.

Another potential risk factor involves predation experience. In the first case, the dolphin was a sexually immature animal. In the stomach several smaller squids were found together with the very large undigested squid body whose tentacles were protruding from the mouth. It might be possible that the last prey was too large for his capability. Therefore, age/experience might have played a role.

The second case involved a sexually mature female. It presented a poor body condition, chronic multiorganic parasitosis, and unilateral pneumothorax. We believe pre-existing disease processes, especially pneumothorax, might have predisposed to develop a decompression pathology in this animal. Pneumothorax would increase the floatability of the dolphin and modify the depth for lung collapse. Spontaneous pneumothorax can develop and turn into fatal tension pneumothorax^34^. Pneumothorax is considered to preclude diving in human divers^34^.

In this study, both animals likely experienced a stress response to the prey interaction. Stress responses, understood as a helpful set of homeostatic mechanisms for the survival of the species, comprises respiratory and circulatory events, and biochemical and haematological changes^34^. Thus, a stress response could have compromised the dive response, as well as the dive behaviour. Extreme or prolonged stress responses can potentially compromise the survival chance of the affected animals. Most mammals, including cetaceans, share the stress response, but it can differ markedly between species and individuals according to differences in physiology, hormonal status and previous experiences^37^.

Many of the physiological changes experienced by these dolphins remain unknown. Further investigations should be carried out to understand how stressful situations such a battle with a large squid (during hunting) or exposure to anti-submarine mid-frequency active sonar, may affect the dive profile or the dive response (e.g., heart rate, blood pressure, peripheral vasoconstriction) of healthy or diseased dolphins, in order to drive it over a non-reversible condition leading to death.

Summarizing, we present two cases of Risso’s dolphins with pathological findings and gas analysis consistent with acute lethal decompression sickness and with an absence of pathological and epidemiological evidence of other deadly systemic, inflammatory, infectious, or neoplastic diseases, or human interaction (e.g.: ship collision, fishing interaction, military sonar…), but with evidence of a recent fight with a squid and sudden death. Thus, the present results support predation (squid hunting) as the most likely cause of these two Risso’s dolphins diving fatalites. Dolphin individual predisposing risk factors resembling those described in human divers are suspected and merit further investigation.

## Material and Methods

From 2000 to 2015, 506 cetaceans stranded and died or died and stranded in the Canary Islands (Spain) for unknown reasons. Systematic pathological studies were performed on the carcasses to find out the cause of death and/or stranding. Required permission for the management of stranded cetaceans was issued by the environmental department of the Canary Islands’ Government and the Spanish Ministry of Environment. No experiments were performed on live animals. For this study, we have excluded 13 animals that stranded in temporal and spatial association with military exercises since these cases have already been published^4,16^. Therefore 493 animals were considered within the study period. This study focuses on two out of 12 Risso’s dolphins that stranded in the Canary Islands (Spain) (Table 1).

The conservation of the carcass was evaluated using a score from 1–5 following Kuiken & García-Hartmann (1991) and Geraci and Lounsbury (2005) with a small modification: code 1 was a very fresh carcass (post-mortem time less than 12 hours). All animals were necropsied following a standardized protocol^38^.

Since 2007, specific protocols were developed and carried out to study gas embolism. For the estimation of the abundance of gas bubbles a gas score was calculated retrospectively using pictures and descriptions of the necropsy report^8^. This gas score consists in describing the presence and abundance of gas bubbles in different vascular and extravascular locations (n = 5). The gas score scale used was: 0 for absence of gas bubbles, 1 for the presence of few-moderate gas bubbles, and 2 for abundant presence of gas bubbles. Gas score at the different locations were summed to obtain a new total gas score with a scale from 0–10. Because gas score was done retrospectively, information from one or more locations were missing occasionally. Those animals with information missing from more than one location were not included in the study. Those that had information missing in one location were marked with a star, to indicate that the total gas score of those animals might be up to two grades higher. Gas score was calculated in 10 out of 12 Risso’s dolphins (Table 1).

Gas sampling and analysis were performed following Bernaldo de Quirós *et al*. (2011 and 2012)^39,40^. Briefly, intravascular gas bubbles were extracted using disposable insulin syringes (BD Plastipak U-100 insulin) and its content was promptly injected into a 5-mL additive-free vacutainers®. Gas form the intestines was collected directly with the vacutainers®. The gas mixed with the blood inside the heart was retrieved using an aspirometer (U201100896). The aspirometer separated the gas from the blood. Gas samples were stored in vacutainers® at room temperature. Gas composition was analysed using a gas chromatograph (Varian 450-GC) equipped with a Varian CP7430 column and a thermal-conductivity detector (TCD) and a flame-ionization detector (FID) disposed one after the other. The presence and abundance of gas bubbles limited the number of animals from which gas samples could be collected. Gas sampling and analysis was performed in three Risso’s dolphins.

Tissues from most organs were collected and fixed in 10% buffered formalin and processed for routine light microscopy. Additionally, lung tissues were post-fixated with OsO_4_ before being paraffin-embedded, for fat emboli detection^4^.

Samples for microbiological studies (skin with blubber, skeletal muscle, lung, liver, kidney, spleen, brain, and other selected organs) were stored at −80 °C. Routine microbiological studies were performed^32^. Those samples were additionally checked by PCR for CeMV (Cetacean Morbillivirus)^41^ and Herpesvirus^42^.

### Data availability statement

We have provided in the manuscript all the necessary data to support our results. If referees consider any more data is necessary we will be happy to provide it in the revised manuscript.
